# Conductivity tensor of graphene dominated by spin-orbit coupling scatterers: A comparison between the results from Kubo and Boltzmann transport theories

**DOI:** 10.1038/srep23762

**Published:** 2016-03-31

**Authors:** Zhe Liu, Liwei Jiang, Yisong Zheng

**Affiliations:** 1 Key Laboratory of Physics and Technology for Advanced Batteries, College of Physics, Jilin University, Ministry of Education, Changchun 130012, China

## Abstract

The diagonal and Hall conductivities of graphene arising from the spin-orbit coupling impurity scattering are theoretically studied. Based on the continuous model, i.e. the massless Dirac equation, we derive analytical expressions of the conductivity tensor from both the Kubo and Boltzmann transport theories. By performing numerical calculations, we find that the Kubo quantum transport result of the diagonal conductivity within the self-consistent Born approximation exhibits an insulating gap around the Dirac point. And in this gap a well-defined quantized spin Hall plateau occurs. This indicates the realization of the quantum spin Hall state of graphene driven by the spin-orbit coupling impurities. In contrast, the semi-classical Boltzmann theory fails to predict such a topological insulating phase. The Boltzmann diagonal conductivity is nonzero even in the insulating gap, in which the Boltzmann spin Hall conductivity does not exhibit any quantized plateau.

As an effective continuous model, a massless Dirac equation can well describe the low-energy electron structure of graphene^1^,^2^,^3^. This indicates that the low-energy electrons in graphene behave like chiral fermions^4^,^5^. In addition, as the energy reduces to the Dirac point (the zero-energy point), the electronic wavelength increases rapidly and diverges ultimately. As a result, some quantum transport features occur unique to graphene, such as the weak-antilocalization^6^,^7^ and the constant conductivity in the long wavelength limit^8^. Of course, these features of graphene conductivity can not be described by semi-classical Boltzmann transport theory which completely excludes the quantum interference effect.

The invalidity of Boltzmann theory to describe the low-energy electronic transport properties in graphene was verified in previous literature. For example, theoretical studies about the conductivity based on the Kubo formula, a quantum transport theoretical framework, reported that graphene conductivity at the Dirac point is of the order of *e*^2^/*h* as long as one of the chiral symmetries of graphene is preserved^3^,^9^. Such a theoretical prediction was demonstrated by subsequent experiments^5^,^10^. However, the Boltzmann transport theory fails to predict such an interesting property of graphene, though it is often employed to study the electronic transport properties of conventional semiconducting and metallic materials.

Apart from the aforementioned intra-valley and inter-valley scattering impurities which are irrelevant to the electronic spin degree of freedom, another type of impurity, the spin-orbit coupling (SOC) impurity in graphene comes into being under up-to-date experimental circumstances^11^,^12^,^13^. For example, when heavy adatoms such as indium and thallium are adsorbed on the graphene sheet, they appear to form randomly distributed clusters with strong extrinsic SOC. Unlike ordinary scatterers, the SOC impurity plays kind of a dual role in graphene. On the one hand, it enhances the intrinsic SOC interaction in graphene effectively, and drives graphene into a quantum spin Hall (QSH) state^14^,^15^,^16^, which is a topologically nontrivial phase^17^,^18^,^19^. On the other hand, it is still a scatterer due to its random nature to affect the electronic transport to some extent^20^,^21^,^22^. Therefore, to clarify the effect of the SOC impurity on the transport properties of graphene, the conductivity was calculated in relevant works with different theoretical approaches including the Boltzmann theory^20^,^21^, the Landauer-Büttiker formula^11^,^12^,^13^ and the Kubo-Bastin formula^22^. There are also theoretical works based on Dirac equation to study the QSH state in graphene^23^,^24^,^25^,^26^. However, in those works, the SOC interaction is in the band and does not act as scatterers. As mentioned above, in the presence of ordinary scatterers, the Boltzmann transport theory is invalid in the low-energy region due to the exclusion of the quantum interference. However, the SOC impurity is expected to open a band gap in the low-energy region, although no signature of induced SOC gap has been observed in experiments so far^27^,^28^,^29^. Therefore, whether such a semi-classical approach is suitable to describe the SOC impurity dominated electronic transport in graphene deserves a detailed check. The Landauer-Büttiker formalism is a quantum transport theory, but it is suitable to calculate the conductance of a particular finite-size mesoscopic structure which is coupled to leads. It is an awkward approach to calculate the conductivity tensor of a disordered two-dimensional infinite system. In a previous work^22^, we have calculated the conductivity tensor of graphene with SOC impurities by Kubo-Bastin formula based on a graphene-only tight-binding (TB) model^12^ using Chebyshev expansion^30^. However, we can only obtain numerical results, and the numerical calculations are relatively time-consuming since a finite-size but large enough sample which is subjected to periodic boundary conditions is needed to simulate a two-dimensional bulk system.

In such a situation, in this work, we address the conductivity tensor of graphene arising from the SOC impurity scattering by means of Kubo-Středa formula. We derive analytical expressions about the diagonal and Hall conductivities within self-consistent Born approximation (SCBA)^3^,^31^, which can give reasonable results in the case of weak scattering. We also present a Boltzmann theoretical treatment of the same conductivity tensor. Our purpose is to give a detailed comparison between the conductivity tensors obtained by the quantum and the semi-classical transport theories. It turns out that the results from Kubo formula exhibit an insulating band gap around the charge neutral point for diagonal conductivity and a quantized spin Hall plateau in the gap, indicating that the SOC impurities drive graphene to a QSH state. However, these features are absent in the results from Boltzmann theory. The Boltzmann diagonal conductivity is nonzero even in the insulating gap. Moreover, the spin Hall conductivity from Boltzmann theory does not exhibit any quantized value.

## Results

### The Model Hamiltonian and the Green Function

In pristine graphene, the low-energy electron in *K* or *K*′ valley can be well described by a massless Dirac Hamiltonian^2^,^3^, which takes a form as


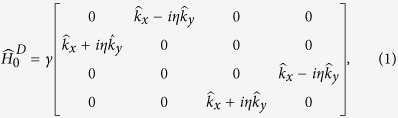


where the band parameter 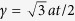 with *a*(≈2.46 Å) being the lattice constant of graphene and *t*(≈3 eV) the hopping energy between the nearest-neighbor carbon atoms. 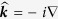 is the wavevector operator in real space. *D* labels the *K* or *K*′ valley and *η* = +1(*η* = −1) for *D* = *K*(*D* = *K*′). The corresponding four-dimensional spinor wave function has the form 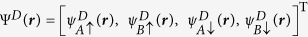, where *A*/*B* represents the sublattice and ↑/↓ represents the electronic spin. According to relevant literature, the electronic SOC interaction in graphene can be incorporated into such a low-energy theoretical framework by adding a mass term to the above Dirac Hamiltonian^26^. Therefore, to model *N*_*i*_ randomly distributed SOC scatterers in graphene, we can assume that the scattering potential takes a form as


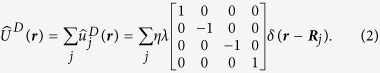


It gives a Dirac mass in the electronic spin and sublattice space and takes the form of delta function in the coordinate space. *λ* stands for the SOC potential strength. ***R***_*j*_ denotes the central position of an individual SOC impurity. It captures the SOC effect of the randomly distributed clusters of heavy adatoms on graphene sheet in an actual experimental situation. Noting that the delta function potential is only appropriate to the limiting case of the cluster size being far smaller than the electronic Fermi wavelength. It becomes invalid as the cluster size gets very large. In a recent literature^13^, it was found that large cluster size can destroy the QSH state of graphene. To study such an effect, we need to choose other function forms to mimic the finite range scattering potential in real space, e.g. the Gaussian function. It is an interesting issue, and could be studied in a future work.

Next we will introduce the Green function and the corresponding self-energies, in terms of which the DOS and the conductivity tensor of graphene in the presence of SOC scatterers are formulated. Prior to proceed, one can notice from the above Hamiltonian that the different valley and spin states are actually decoupled. Therefore, we can restrict our theoretical treatment in the *K* valley and spin-up subspace. The results in other subspaces can be derived similarly. For a spin-up electron in *K* valley, the unperturbed Hamiltonian is


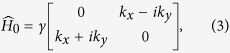


and the scattering potential reduces to


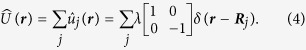


The eigenstate of the unperturbed Hamiltonian eq. (3) can be easily obtained, it is given by


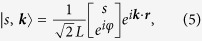


where *s* = +1(*s* = −1) denotes the conduction (valence) band, *L* is the linear size of the graphene sheet and *φ* is the angle between ***k*** and positive *x* axis, i.e. tan *φ* = *k*_*y*_/*k*_*x*_. The corresponding eigen energy is 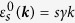.

In the eigen representation of 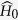, the matrix element of the Green function of the system is defined as


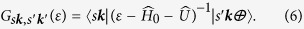


For evaluating the macroscopically observable physical quantities, such as the conductivity tensor, we need to perform an average of the Green functions over all possible SOC impurity configurations, i.e. 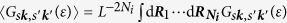. Such an averaged Green function is connected to the proper self-energy Σ_*s**k***,*s*′***k***'_(*ε*) by Dyson equation


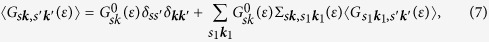


where 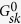 is the unperturbed Green function


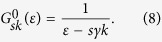


**Figure 1:**
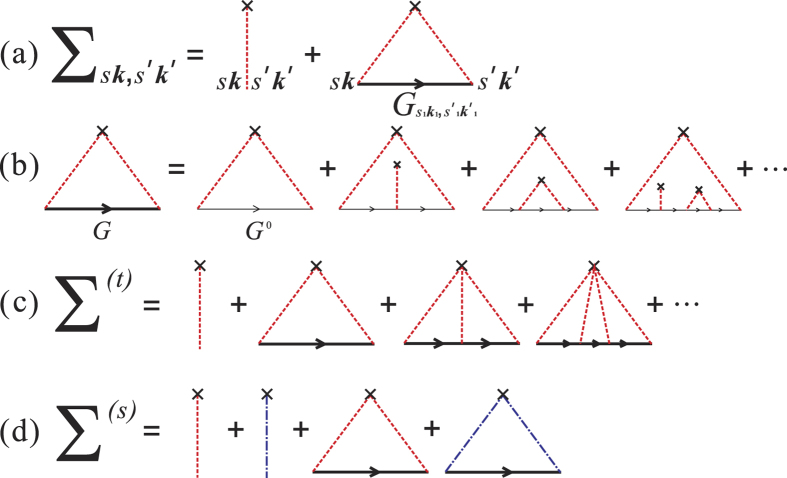
Feynman diagrams in the calculations. (**a**) The SCBA self-energy. (**b**) The decomposition of the second term in (**a**) into terms including only unperturbed Green functions. (**c**) The self-energy under t-matrix approximation. (**d**) The SCBA self-energy in the presence of usual scalar scatterers. The bold and thin lines denote the perturbed and free Green functions, respectively. The dashed lines and dash dot lines indicate the SOC and usual scalar scattering potentials, respectively. The crosses denote the configuration average of the impurities.

Within SCBA, the self-energy in eq. (7) obeys an equation as follows


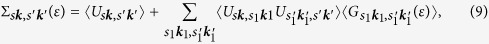


which can also be depicted by the Feynman diagrams shown in Fig. 1(a). In eq. (9), 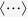 means the impurity configuration averaging to the quantity in it, in the same way as explained above to the Green function. As a result of the impurity configuration averaging, the Green function 〈*G*_*s**k***,*s*′***k***′_(*ε*)〉 is diagonal and isotropic about ***k***, which can be seen by expanding the second diagram in Fig. 1(a) in terms of diagrams including only unperturbed Green functions, as shown in Fig. 1(b). Thus, we can use the shorthand *G*_*ss*′*k*_(*ε*) ≡ 〈*G*_*s**k***,*s*′***k***′_(*ε*)〉. Similarly, the self-energy is also ***k***-diagonal. Moreover, it is independent of ***k***. Up to now, the self-energy reduces to


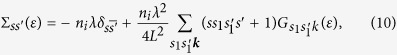


where *n*_*i*_ = *N*_*i*_/*L*^2^ is the concentration of the SOC impurities. To be more specific, we can define the even and odd self-energies as


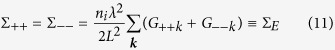



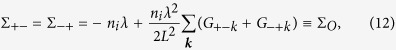


where the argument *ε* of the self-energies is dropped for simplicity. With the help of Dyson eq. (7), we can express the Green functions above in terms of the self-energies. Consequently, we get the self-consistent equations about the self-energies. They are


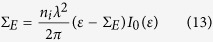



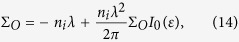


where *I*_0_ denotes the integral


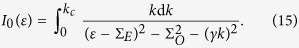


More details about the derivation of eqs (13) and (14) are given in the Methods Section. We can take advantage of eqs (13) and (14) to evaluate the self-energies by numerical iteration method. When evaluating the integral *I*_0_ numerically, we need to choose a cut-off upper limit *k*_*c*_. The choice of this cut-off wavevector is somewhat arbitrary, but the results depend only weakly on *k*_*c*_ when it is large enough. When formulating the Hall conductivity given below, the derivatives of the self-energies with respect to the energy argument *ε* are involved. Differentiating eqs (13) and (14), we can get the equations about the derivatives of the self-energies


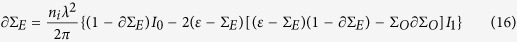



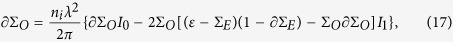


where the shorthands ∂Σ_*E*/*O*_ ≡ (∂Σ_*E*/*O*_/∂*ε*) are adopted. *I*_1_ is another *k*-integral similar to *I*_0_, which is given by


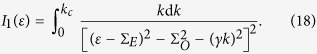


In general, the self-energies are complex functions which depend on a complex energy argument. In particular, we can denote the retarded and advanced self-energies as


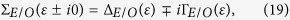


where Δ_*E*/*O*_ and Γ_*E*/*O*_ are real functions of the real energy argument. Hence, the density of states (DOS) can be expressed in terms of the imaginary part of the even self-energy


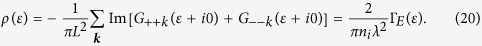


In the weak scattering limit, namely *n*_*i*_*λ* → 0, one can readily find that Σ_*E*_ → 0 and Σ_*O*_ → −*n*_*i*_*λ*. Accordingly, the DOS has the following approximation


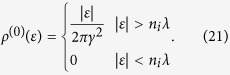


This implies that within SCBA, the system has an energy gap which is proportional to the concentration and the strength of the SOC scatterers to the weak scattering limit. This gap is expected to be topologically nontrivial because it is opened by the SOC impurity. A more detailed derivation of eq. (21) is also given in the Methods Section.

### The Conductivity Tensor from Kubo Formalism

To work out the expressions of the conductivity tensor, i.e. the diagonal and Hall conductivities, we start from the Kubo-Bastin formula for noninteracting electrons^32^


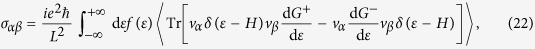


where *α* or *β* denotes an arbitrary Cartesian coordinate *x* or *y*, and *G*^±^ = *G*(*ε* ± *i*0) is the retarded/advanced Green function. The delta functions can be expressed in terms of Green functions, i.e. *δ*(*ε* − *H*) = −*π*^−1^ Im *G*(*ε* + *i*0). Following the steps in ref. 33, we can simplify the expressions of diagonal and Hall conductivities at zero temperature. As a result, the diagonal conductivity depends only on the Fermi energy *ε*_*F*_ and can be written as


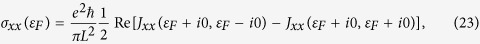


which is equivalent to Kubo-Greenwood formula^34^ at zero temperature. However, the Hall conductivity is divided into two terms. One term depends only on the Fermi energy, whereas the other term is an integral up to the Fermi energy. Therefore, the Hall conductivity has the form


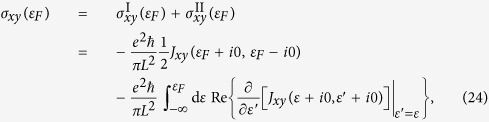


which is equivalent to Kubo-Středa formula^35^. In eqs (23) and (24), the correlation function *J*_*αβ*_(*ε*, *ε*′) is defined as


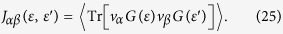


**Figure 2:**
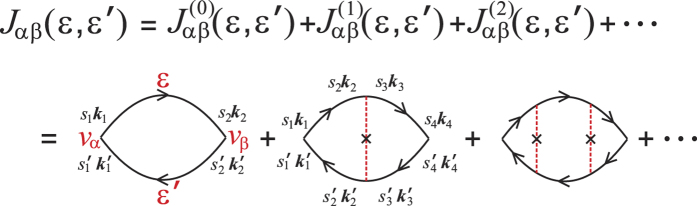
Feynman diagrams of the correlation function *J*_*αβ*_ (*ε*, *ε*′) within SCBA.

The velocity operator *v*_*α*_ can be represented by Heisenberg equation *v*_*α*_ = [*H*, *α*] = (*γ*/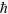)*σ*_*α*_. In general, *J*_*αβ*_ is a complex function of complex energy arguments *ε* and *ε*′. Within SCBA, the correlation function *J*_*αβ*_ (*ε*, *ε*′) can be obtained by summing the Feynman diagrams including the vertex corrections, as shown in Fig. 2. The summation turns out to be the sum of a geometrical series, and the results for *J*_*xx*_ and *J*_*xy*_ are


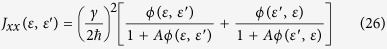


and


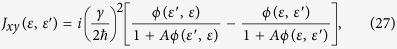


where *A* = *n*_*i*_*λ*^2^/4*L*^2^, and the function *ϕ* is


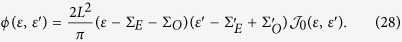


In eq. (28), Σ_*E*/*O*_ and 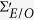 refers to Σ_*E*/*O*_(*ε*) and Σ_*E*/*O*_(*ε*′), respectively, and 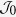 represents the integral


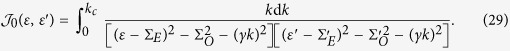


Substituting eqs (26, 27, 28, 29) into eqs (23) and (24) and taking advantage of the self-consistent eqs (13, 14, 15, 16, 17), we can express the diagonal and Hall conductivities in terms of the self-energies and the derivatives of them. Therefore, the conductivity tensor can be calculated directly from the self-energies and the derivatives of them, which can be calculated by self-consistent iteration method. For more details about the derivation of the conductivity tensor, see the Methods Section. We only give the result of the diagonal conductivity to the weak scattering limit here


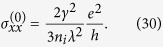


This result can also be obtained via the semi-classical Boltzmann transport theory, as can be seen in the following section.

### The Conductivity Tensor from Boltzmann Theory

In this section, we derive the expression of diagonal and Hall conductivities from Boltzmann transport theory. We start from the semi-classical conductivity formula


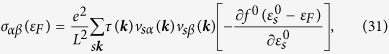


where *f *^0^ is the Fermi distribution function of the equilibrium state. ***v***_*s*_(***k***) is the group velocity of the electronic wave packet 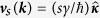, with 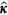 being the unit vector along the ***k*** direction. The relaxation time *τ* in eq. (31) can be deduced from Boltzmann equation


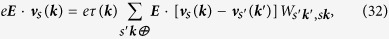


where ***E*** is an external electric field. *W*_*s*′***k***′,*s**k***_ is the scattering rate from |*s*, ***k***〉 to |*s*′, ***k***′〉 and can be written in terms of the scattering matrix element as follows


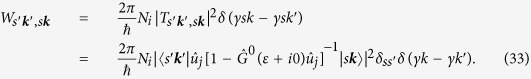


The scattering matrix element *T*_*s*′***k***′,*s**k***_ can be expanded as a geometrical series. After summing up the series, the scattering matrix element becomes


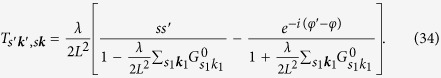


The summation of the unperturbed Green functions yields


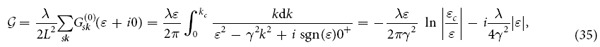


where *ε*_*c*_ = *γk*_*c*_. Substituting eqs (34) and (35) into eq. (33), and dropping off the terms which are of the second or higher order of *λ*, we get the scattering rate


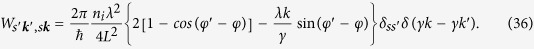


Substituting this scattering rate into eq. (32), we obtain the inverse relaxation time


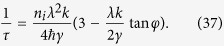


Substituting eq. (37) into eq. (31), we can obtain the expressions of the diagonal and Hall conductivities. The first term in eq. (37) can give a constant value of the diagonal conductivity


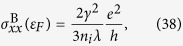


which is the same as the weak scattering limit of the Kubo diagonal conductivity given by eq. (30). However, to get a nonzero Hall conductivity, we must include the second term of the inverse relaxation time, which corresponds to skew scattering^20^,^21^. The resultant Hall conductivity is


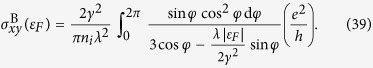


So much for the analytical results. We will discuss the numerical results in the next section.

## Discussion

**Figure 3:**
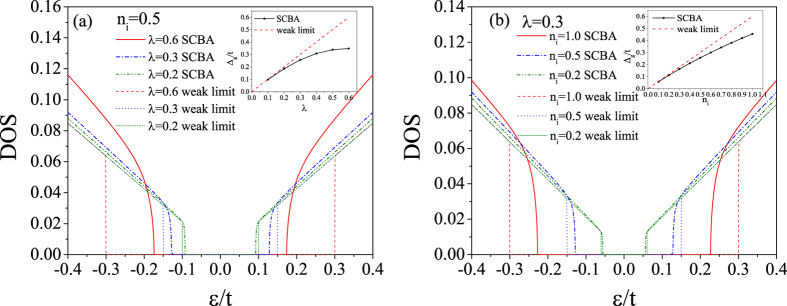
The DOS calculated by SCBA. (**a**) For *n*_*i*_ = 0.5 and different impurity strengths. The insight shows the energy gap Δ_*g*_ as a function of the impurity strength *λ* for *n*_*i*_ = 0.5. (**b**) For *λ* = 0.3 and different impurity concentrations. The insight shows the energy gap Δ_*g*_ as a function of the impurity concentration *n*_*i*_ for *λ* = 0.3.

**Figure 4:**
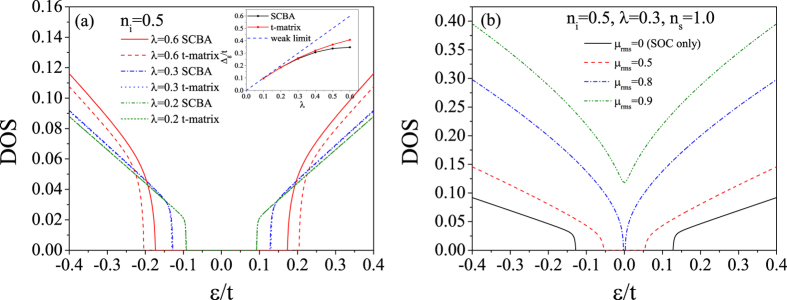
(**a**) A comparison between the DOS calculated by SCBA and t-matrix approximation for *n*_*i*_ = 0.5 and different impurity strengths. The insight shows the energy gaps obtained via these two methods as functions of the impurity strength for *n*_*i*_ = 0.5. (**b**) The DOS calculated by SCBA in the presence of usual scalar scatterers for *n*_*i*_ = 0.5, *λ* = 0.3, *n*_*s*_ = 1.0 and different strengths of the scalar scattering potential.

With the formulation of the conductivity tensor developed in the preceding section, we are in a position to perform a numerical calculation of the conductivity spectrums, i.e. the diagonal and the Hall conductivities as functions of the Fermi energy. And then a comparison between the results from Kubo and Boltzmann theories can be made, based on which the validity of the semi-classical Boltzmann transport theory to describe the conductivity tensor of graphene arising from SOC impurities can be clarified. However, before doing this, we would like to present numerical results about the DOS spectrums. From the approximate result of DOS to the weak scattering limit eq. (21), we know that the SOC impurity can open a band gap which is proportional to the product of the scatterer strength and concentration. The numerical results of DOS spectrums are shown in Fig. 3 for different strengths and concentrations of the SOC impurities. We can clearly see that a gap occurs around the Dirac point (zero-energy point) for all the cases. And at relatively weak strength and concentration of the SOC impurities, the approximate expression of DOS to the weak scattering limit, i.e. eq. (21), can give satisfactory results, well agreeing with the numerical results within SCBA. In the insights of Fig. 3, we give the dependence of the band gap on the strength and concentration of the SOC impurities. As given by eq. (21), the gap is 2*n*_*i*_*λ* in the weak scattering limit. The numerical results shown in the insights of Fig. 3 indicate that such a simple relation holds true only in the case of relatively weak scattering. However, some previous works reported that the simple linear dependence of the band gap on the product of the SOC scatterer strength and concentration, i.e. Δ_*g*_ = 2*n*_*i*_*λ*, is still a good approximation even when the system is far away from the weak scattering limit^13^,^22^,^36^. The deviation of the band gap from the linear relation as shown in the insights of Fig. 3 is due to that the SCBA employed in this work excludes some high-order scattering processes. To illustrate this, we can go one step further to calculate the DOS under t-matrix approximation instead of SCBA. Within t-matrix approximation, we add up all the terms represented by the Feynman diagrams shown in Fig. 1(c) to obtain the self-energy self-consistently. The results are shown in Fig. 4(a). We can see that when the impurity strength is relatively weak (*λ* < 0.4), the t-matrix approximation and the SCBA almost give the same result. The band gap as a function of the SOC impurity strength obtained by t-matrix approximation is closer to the linear rule than that from SCBA, as seen in the insight of Fig. 4(a). Another issue we would like to study is the effect of usual scalar scatterers on the topologically nontrivial gap. To derive the SCBA self-energy in this case, we need to evaluate the Feynman diagrams shown in Fig. 1(d). We assume that the strength of the scalar scatterers has a zero value on average. Thus, we characterize the disorder by the root mean square strength 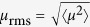. The concentration of the scalar scatterers is denoted by *n*_*s*_. The DOS results are shown in Fig. 4(b). It can be readily seen that the band gap opened by the SOC scatterers survive a weak scalar disorder. However, a strong enough scalar disorder will close the gap, and hence destroy the QSH state. The derivation of the self-energy within t-matrix approximation and the SCBA self-energy in the presence of usual scalar scatterers are given in the Methods section.

**Figure 5:**
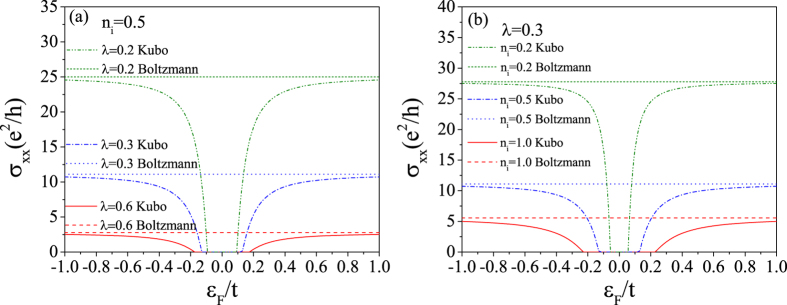
The diagonal conductivity of *K* valley spin-up electrons as a function of the Fermi energy calculated by both the Kubo formula and the Boltzmann equation. (**a**) For *n*_*i*_ = 0.5 and different impurity strengths. (**b**) For *λ* = 0.3 and different impurity concentrations.

Now, we turn to discuss the numerical results about the conductivity tensor. The diagonal conductivity spectrums are shown in Fig. 5 for different SOC scatterer strengths and concentrations. From this figure, we can see that the result from Boltzmann theory is almost a nonzero constant even in the gap. It comes to the conclusion that the semi-classical Boltzmann theory fails to describe the electronic transport properties of graphene arising from the SOC scattering not only in the vicinity of the Dirac point, but also around the band edges. Notice that when the band gap is large, the band edges are far away from the Dirac point. As shown in Fig. 5(a,b), *σ*_*xx*_ from Kubo formula vanishes in the SOC band gap. It increases gradually and tends to the Boltzmann result as the Fermi energy goes away from the gap. This result tells us that the Boltzmann theory is only valid in the short electronic wavelength regions where the quantum interference effect becomes weak. Before ending the discussion about the diagonal conductivity spectrum, we would like to point out that the electronic contributions to the diagonal conductivity from other subband spaces, e.g. the spin-down electrons or *K*′ valley electrons, are exactly the same as shown in Fig. 5.

**Figure 6:**
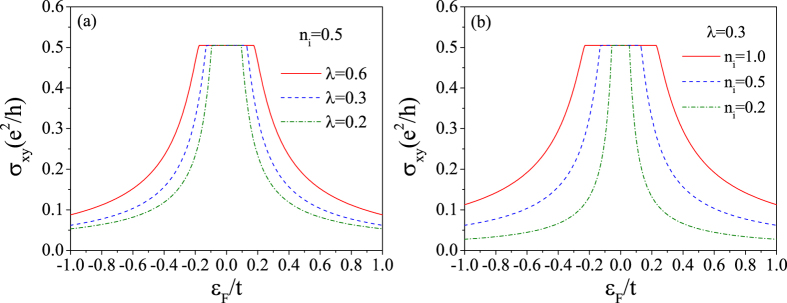
The Hall conductivity of *K* valley spin-up electrons as a function of the Fermi energy calculated by Kubo formula. (**a**) For *n*_*i*_ = 0.5 and different impurity strengths. (**b**) For *λ* = 0.3 and different impurity concentrations.

**Figure 7:**
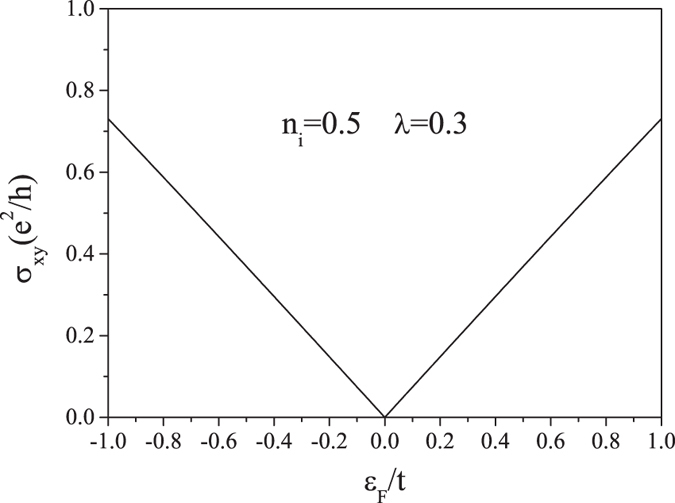
The Hall conductivity of *K* valley spin-up electrons as a function of the Fermi energy calculated by Boltzmann equation for *n*_*i*_ = 0.5 and *λ* = 0.3.

In Fig. 6, the Hall conductivity spectrums obtained by Kubo formula are shown for different SOC scatterer strengths and concentrations. From this figure, we can see that for the *K* valley spin-up electron, the Hall conductivity shows a plateau with a height of *e*^2^/2*h* within the gap, regardless of the scattering strength and concentration. This result indicates that the gap opened by SOC impurities is topologically nontrivial. Considering that the spin-down electron contributes an opposite Hall conductivity due to the time reversal symmetry, the total Hall conductivity vanishes. However, the system is on a QSH state with a quantized spin Hall conductivity 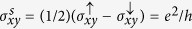. The results shown in Fig. 6 support our previous theoretical prediction that the randomly distributed SOC impurities can drive the graphene into a QSH state^22^. This quantized spin Hall conductivity has been obtained in ref. 23 where the SOC interaction does not act as a scatterer. Now the interesting thing is that the QSH state emerges even if the SOC is in the scatterer but not in the band. From Fig. 6, we can also see that outside the gap, the Hall conductivity is nonzero, though it decreases rapidly as the Fermi energy goes away from the gap. This indicates that in this region, the system is in a spin Hall regime even though the spin Hall conductivity is not quantized. The nonzero spin Hall conductivities outside the gap are also discussed in ref. 23, in which the intrinsic Hall conductivity agrees with our results. A typical numerical result of *σ*_*xy*_ from Boltzmann transport theory is shown in Fig. 7. We can see that the Hall conductivity here is completely different from that calculated from Kubo theory. We cannot observe a spin Hall plateau in the gap region and the spin Hall conductivity tends to zero when *ε*_*F*_ → 0. Although the spin Hall conductivity from Boltzmann theory is not quantized, it has nonzero values especially when the Fermi energy is far away from the Dirac point. This nonzero spin Hall conductivity is the result of skew scattering, which is detailed studied in ref. 21.

In summary, a theoretical study on the conductivity tensor of graphene arising from the SOC impurities is presented in this work. The calculated results of the conductivity tensor treated by the semi-classical Boltzmann and the quantum transport approaches are compared. In the quantum transport approach realized by the Kubo-Středa formula within SCBA, the diagonal conductivity shows an insulating gap around the Dirac point. Meanwhile, in such a gap the spin Hall conductivity shows a well-defined quantized plateau. These features indicate unambiguously the realization of the topologically nontrivial state of graphene driven by the randomly distributed SOC impurities. In contrast, within the Boltzmann theory, the diagonal conductivity does not vanish and the spin Hall conductivity does not show any quantized plateau in the vicinity of the Dirac point. Thus, Boltzmann theory cannot well describe the low-energy transports in graphene arising from the SOC scatterers. Finally, we have to point out that recent experimental works on the transport properties of graphene dominated by heavy adatoms found no evidence of a SOC band gap^27^,^28^. The possible reason might be the clustering effects and the long range Coulomb scattering. Therefore, it is useful and interesting to perform a further theoretical study on the effects of the cluster size and the Coulomb scattering potential on the QSH state.

## Methods

### The Derivation of the Self-Energies

In this subsection, we give the details for the self-consistent equations of the self-energies. We start from the self-energy within SCBA


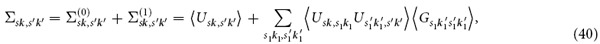


which can be represented by the Feynman diagrams in Fig. 1(a). The first term is


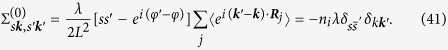


Because the average Green function is diagonal and isotropic about ***k***, the shorthand notation *G*_*ss*′*k*_ is adopted. Therefore, the second term of the self-energy yields


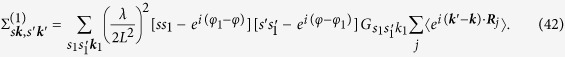


Because the Green function is isotropic about ***k***, all the terms which has an exponential factor like 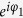 vanishes after the summation of ***k***_1_. Thus,


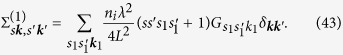


We can see from eqs (41) and (43) that the self-energy is diagonal about ***k*** and does not depend on ***k***. Therefore, the self-energy has the form of eq. (10). Moreover, there are only two different self-energies, the even self-energy Σ_*E*_ and the odd self-energy Σ_*O*_, which are defined in eqs (11) and (12). By Dyson eq. (7), we can express the Green functions in terms of the self-energies, for example


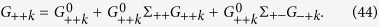


*G*_−+*k*_ can also be evaluated by Dyson equation


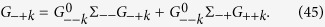


Combining eqs (44) and (45), we can solve *G*_++*k*_ and *G*_−+*k*_. By the same token, we can also solve the other two Green functions. The results are


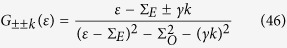



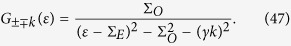


Substituting eqs (46) and (47) into eqs (11) and (12) and transforming the summation into integration, we obtain the self-consistent equations for the self-energies, i.e. eqs (13) and (14).

In the weak scattering limit, we first substitute Σ_*E*_ = 0 and Σ_*O*_ = −*n*_*i*_*λ* into the expression of *I*_0_ but keep the imaginary part of Σ_*E*_ in the denominator


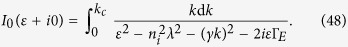


Making a variable substitution *t* = (*γk*)^2^ and *t*_*c*_ = (*γk*_*c*_)^2^, and noticing Γ_*E*_ is a positive infinitesimal quantity, we have


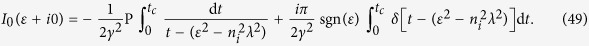


The first term is a principle-value integral, and whether the second term vanishes depends on the sign of 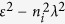. Substituting eq. (49) into the self-consistent equations of the self-energies, we obtain the self-energies to the weak scattering limit


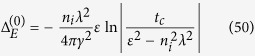



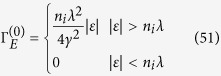



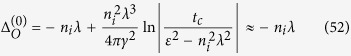



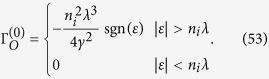


The DOS in the weak scattering limit *ρ*^(0)^ in the main text is obtained directly from 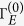. Notice that the four quantities above are of different orders of *n*_*i*_ and *λ*, 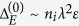, 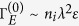, 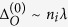 and 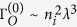. Thus, we take the limits Σ_*E*_/*ε* → 0, Δ_*O*_ → −*n*_*i*_*λ* and Γ_*O*_ → 0 when evaluating the diagonal conductivity to the weak scattering limit, i.e. eq. (30).

Within t-matrix approximation, the self-energy can be expressed by a geometric series which can be represented by the Feynman diagrams shown in Fig. 1(c). A general *n*-order term is


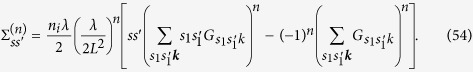


Taking advantage of eqs (46) and (47), the self-consistent equations of the self-energies within t-matrix approximation can be obtained


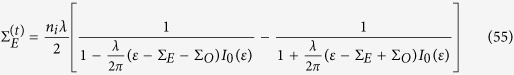



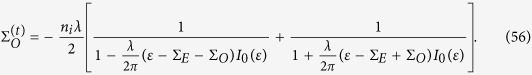


In order to study the effect of a usual scalar disorder on the topologically nontrivial gap, we add the following scalar potential to the Hamiltonian


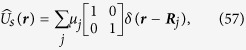


where {*μ*_*j*_} is a set of random numbers to simulate the scalar disorder. We assume 〈*μ*〉 = 0. In this case, we need to evaluate the Feynman diagrams shown in Fig. 1(d). Because of the zero value of the average impurity strength, the second term in Fig. 1(d) is zero. And the fourth term has a similar form as the third term. Therefore, the self-consistent equations of the self-energies within SCBA in this case are


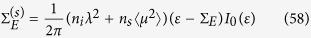



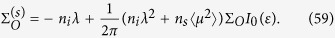


### The Derivation of the Kubo Conductivity Tensor

In this subsection, we give the derivation details of the Kubo conductivities. First, we give the derivation details for the diagonal correlation function *J*_*xx*_(*ε*, *ε*′). The correlation function can be obtained by summing the Feynman diagrams including the vertex corrections, as shown in Fig. 2. The zero-order term is


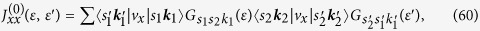


where the summation is over all the band subscripts and wavevectors. The matrix element of *v*_*x*_ yields


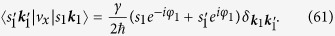


Thus, the velocity operator is diagonal about ***k*** as well as the Green function. Therefore, we let 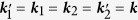 and eq. (60) is transformed to


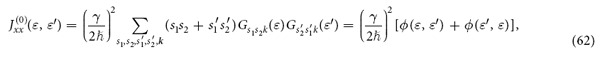


where


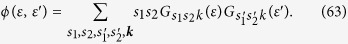


Similarly, due to the diagonal property of the velocity operator and the Green function, in the first-order term we have 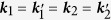 and 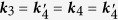 (see Fig. 2). Therefore, the first-order term of the correlation function has the form


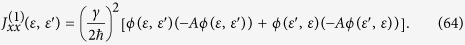


By the same token, the other terms can all be obtained


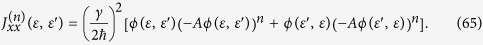


Summing up all the terms, we obtain eq. (26). Equation (27) can be obtained similarly. Transforming the summation in eq. (63) into integration, we obtain eq. (28). When *ε* ≠ *ε*′, the integral 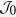 could be simplified as


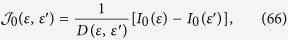


where


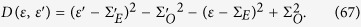


Using the self-consistent equations for the self-energies, we can express the integral *I*_0_ in terms of the self-energies. Hence, the correlation functions can be expressed as


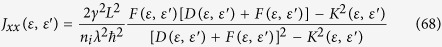



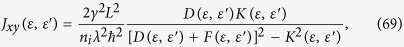


where


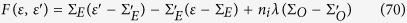



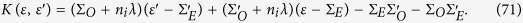


The case when *ε* = *ε*′ is even simpler


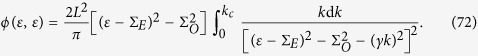


When *k*_*c*_ is large, the integral above converges to a constant and hence *ϕ*(*ε*, *ε*) = −*L*^2^/(*πγ*^2^). Thus,


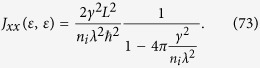


For the special case when the two energy arguments take the values *ε* + *i*0 and *ε* − *i*0, the correlation functions can be expressed by real functions


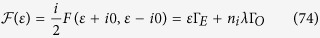



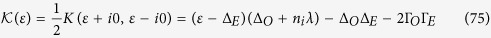



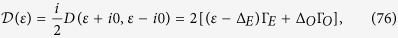


and hence the diagonal conductivity and the first term of the Hall conductivity can be expressed as


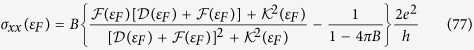



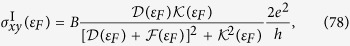


where *B* = *γ*^2^/*n*_*i*_*λ*^2^.

Next, we derive the formula for the derivative of the correlation function. Taking partial derivative with respect to *ε*′ of eq. (27), we get


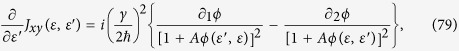


where ∂_1_*ϕ* ≡ (∂/∂*ε*′)*ϕ*(*ε*′,*ε*) and ∂_2_*ϕ* ≡ (∂/∂*ε*′)*ϕ*(*ε*,*ε*′). We calculate ∂_1_*ϕ* first


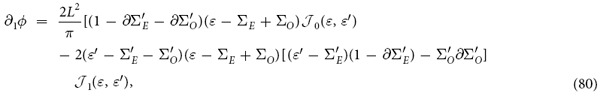


where


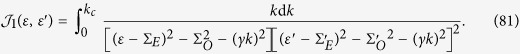


For *ε*′ = *ε*, we have 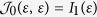 and 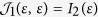 where


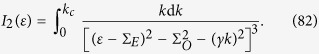


Thus


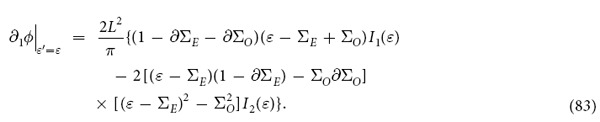


Similarly,


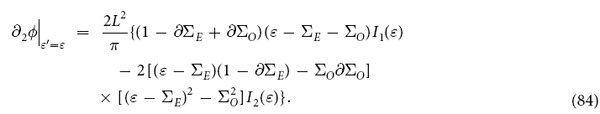


Substituting eqs (83) and (84) into eq. (79), we get


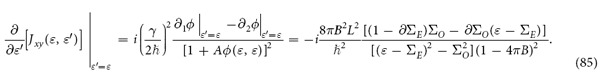


Letting the energy argument in eq. (85) to take the value *ε* + *i*0 and finding the real part, we obtain the expression for the second term of the Hall conductivity


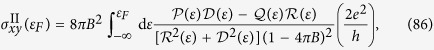


where 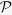, 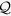, 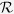 are three real functions as follows


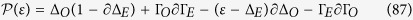



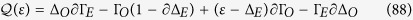



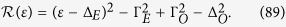


## Additional Information

**How to cite this article**: Liu, Z. *et al*. Conductivity tensor of graphene dominated by spin-orbit coupling scatterers: A comparison between the results from Kubo and Boltzmann transport theories. *Sci. Rep*. **6**, 23762; doi: 10.1038/srep23762 (2016).
